# Prospective and Retrospective Analysis of the Sporting Success of Elite Spanish High and Long Jumpers

**DOI:** 10.5114/jhk/170762

**Published:** 2023-10-11

**Authors:** Pablo Rodriguez-Gomez, Cesar Gallo-Salazar, Juan Jose Salinero

**Affiliations:** 1 Exercise Physiology Laboratory, Camilo Jose Cela University, Madrid, Spain.; 2 Sports Training Laboratory, Faculty of Sports Sciences, University of Castilla-La Mancha, Toledo, Spain.

**Keywords:** age, athletics, jump performance, ranking position, transition rate

## Abstract

The aim of the present study was to analyze the sporting progression from U14 to senior categories of elite Spanish high and long jumpers. For prospective analysis, 300 athletes ranked top 20 at U14 were analyzed (153 female and 147 male). For retrospective analysis, 64 athletes ranked in the top 20 in the senior category were included (21 female and 43 male). Ranking positions were registered in each of the seasons where they presented records. Only 6.3% (19) of athletes who reached the top 20 at U14 became successful senior athletes [4.7% (14) of athletes maintained top 20 status throughout their sporting careers from U14 to senior]. The transition rate from U14 to U16 (35.7%) was the most severe drop down in consecutive categories (after this, it ranged from 47.8 to 66.7%). Of the senior top 20 athletes (64), most of them were already ranked top 20 at U16 (59.4%, 38), at U18 (62.5%, 40), at U20 (70.3%, 45) and at U23 (78.1%, 50). Nevertheless, only 34.4% (22) were top 20 when they were U14 athletes. Transition rates in the top 20 senior athletes ranged from 86.7 to 95.5%. Around one out of four (26.6%, 17) of the top 20 senior athletes maintained top 20 status throughout their sporting careers from U14 to the senior category. Although early success is not a good predictor of senior success, successful senior athletes excelled early on and were able to remain in top rankings throughout their sporting careers of national elite jumpers.

## Introduction

Understanding long-term and sustainable talent development is the key to improving sports planification (Côte et al., 2009; Huxley et al., 2017; Kearney et al., 2021; Kostrzewa et al., 2020). According to Rees et al. (2016) although growth and maturation are important aspects that affect performance at an early age, other aspects, such as the quality of training and competition, family support, injuries and socioeconomic background, have a great influence. There are several ways to achieve elite performance in sports (Huxley et al., 2017), and elite performance is the result of the interaction between genetic and training factors, with both talent identification and management systems being crucial to sporting success (Tucker and Collins, 2012).

Developmental practice patterns leading to rapid adolescent success and long-term senior success seem sometimes conflicting (Bezuglov et al., 2022; Güllich and Emrich, 2014; Siembida et al., 2021). As recently reported, early competitions in the main sport discipline, rapid initial progress and intensive specialized coach-led practice lead to short-term junior-age athletic success, while relatively later participation in the main sport, gradual progress and extensive youth multi-sport practice contribute to long-term adult-age success (Barth et al., 2022). Thus, while youth athletes, coaches and sport managers believe that early sport specialization increases their sporting performance and their chances for later success (Brooks et al., 2018), several studies have reported that early success is not a good predictor for senior success in a wide range of sports (Barreiros et al., 2014; Barth et al., 2022; Bjørndal et al., 2018; Li et al., 2018).

In accordance with this, in track and field athletics, previous research has reported that excelling at the youth level is not a prerequisite for later success (Agudo-Ortega et al., 2023; Boccia et al., 2021c, 2021b; Kearney and Hayes, 2018). However, a recent study from 67,600 athletes (top-100 world ranking in the U18 and U20) showed that the most successful youth athletes were significantly more likely to succeed as senior athletes than their less successful peers (Bezuglov et al., 2022). Therefore, analysis of the transition rate from youth to senior categories and performance progression might be interesting for coaches and sport managers. Previous studies haveanalyzed the trajectory of top-level Italian athletes and found that less than 25% of elite U14 athletes (except women long jumpers, where this value was 38%) maintained their status in adulthood (Boccia et al., 2017, 2019). In the same way, in the United Kingdom, a minority of U15 athletes (10% of males and 25% of females) retained their top-20 status even at the U20 category (Kearney and Hayes, 2018a). More recent research focused on older stages (i.e., U18 athletes ranked in world top 100), showed that only 23.5% entered the top 100 as senior athletes (Bezuglov et al., 2022). Around 20% of the top 50 world U18 sprinters remained in the top 50 in adulthood (Boccia et al., 2021c) and most of the early successful U18 world-class high and long jumpers did not manage to maintain the same status in adulthood (Boccia et al., 2021a).

The path that takes a young athlete to become an adult champion is not linear and is characterized by a remarkable amount of uncertainty (Barth et al., 2022; Hollings et al., 2014; Issurin, 2017; Rees et al., 2016; Spieszny et al., 2022). However, recent research has been also oriented toward a prospective way, showing that senior world-class athletes did not have greater initial potential than their national-class peers. While the latter depleted a large part of their initial potential during junior age, the former depleted a smaller part of it at this stage (Barth et al., 2022). In world-class jumpers, Boccia et al. (2021b) found that the performance progression of senior athletes ranked top 50 and those who failed to be top 50 in the senior category despite being ranked top 50 in the U18 category, was largely indistinguishable up to 19 years of age. In the same way, in the United Kingdom, examining a wide variety of athletics disciplines, weak to moderate correlations between performances at different ages were found until at least U17–U20. Thus, current literature shows that early success is not a prerequisite for senior success and, therefore, the transition rate from junior to senior is low and performance at early age is a weak predictor of adult performance. However, there is still no solid evidence in this field, as most current research has focused only on categories from U18 to senior (Bezuglov et al., 2022; Boccia et al., 2021c, 2021b; Pizzuto et al., 2017), or from U13 to U20 (Kearney and Hayes, 2018), and thus, studies encompassing overall sporting career are scarce (Boccia et al., 2017, 2019). Therefore, the aim of the present study was to analyze the sporting progression from U14 to senior categories of elite Spanish high and long jumpers.

## Methods

### Design

A longitudinal descriptive design was used, based on public data (e.g., age, category, performance and ranking position) available on the website of the Royal Spanish Athletics Federation (RFEA; www.rfea.es; accessed on 03 March 2022).

### Participants

For prospective analysis, 300 athletes ranked top 20 at the U14 category were analyzed, of which 146 athletes competed in the long jump (74 women and 72 men) and 154 athletes in the high jump (79 women and 75 men). For retrospective analysis, 64 athletes ranked in the top 20 in the senior category were included (12 female and 22 male long jumpers; and 9 female and 21 male high jumpers).

### Procedures

#### Prospective Analysis

All athletes ranked within the top 20 in U14 between the 2004/2005 and 2008/2009 athletic seasons were identified. All ranking data for the following seasons were downloaded to be tracked until the 2021 season (this temporary period ensures the follow-up of athletes up at least to their first 3 years of the senior category).

#### Retrospective Analysis

All senior athletes ranked within the top 20 between the 2016/2017 and 2021 seasons were identified. Those with age such that their ranking could be tracked up to U14 were included. Sixty-four athletes were included, of which 19 had already been included in the prospective analysis, and therefore we included 45 new athletes.

Following previous research (Kearney and Hayes, 2018a), a top 20 ranking criterion was chosen as it allowed to include athletes who could reasonably be expected to make the national semifinals. The ranking position was recorded for each athlete and for each season if the athlete was still ranked. Long and high jump are the only events in athletics where there are no regulatory restrictions/changes throughout the different age categories. In Spain, athletic competitions are organized in two (U14, U16, U18 and U20), or three-year (U23) age bands, while the senior band includes any athlete 23 or older. The transition rate was calculated following previous studies (Boccia et al., 2021b) as the percentage of athletes who were top 20 across different categories. For prospective analysis, athletes who first appeared in the top 20 U14 ranking were counted as they remained in the top 20 status in subsequent older categories. For retrospective analysis, athletes included in the top 20 senior rankings were counted as they were already in the top 20 status in previous younger categories. The data were based on publicly available resources, so informed consent and local Ethics Committee approval were not required.

### Statistical Analysis

Data are shown as relative (absolute) frequencies for the data of athletes who appeared/remained in the different categories and contingency tables were used with the McNemar test to analyze the proportion of athletes in/out of the top 20 status in consecutive categories. All statistical analysis was performed with IBM SPSS 28.0 (IBM Corp., Armonk, NY, USA). The level of significance was established at *p*< 0.05 in all cases.

## Results

Table 1 shows the proportion of athletes (out of 300) who, being in the top 20 at the U14 category, appeared again in subsequent categories ranked in the top 20. Prospective analysis revealed that only 6.3% (19) of athletes who reached the U14 top 20 maintained that status at the senior level. Indeed, in the following category (U16), only 35.7% (107) remained in the top 20. Proportion of athletes ranked top 20 dropped progressively to 23% (69) at U18, 12% (36) at U20 and 9% (27) at U23 categories. Change of the status between categories was significant from U16 to U18 (*p*< 0.001), from U18 to U20 (*p*< 0.001) and from U23 to senior (*p* = 0.021). The transition rate from U14 to U16 (35.7%) was the most severe drop in subsequent categories. After this, transition rates from U16 to U18 were 55.1%, from U18 to U20 47.8%, from U20 to U23 61.1% and from U23 to the senior category 66.7%. Finally, only 4.7% (14) of athletes maintained top 20 status throughout their sporting careers from U14 to the senior category.

**Table 1:** Jumpers ranked top 20 at the U14 category who were ranked top 20 in subsequent categories.

	U14	U16	U18	U20	U23	Senior
Top20	300	35.7% (107)	23.0% (69)*	12.0% (36)*	9.0% (27)	6.3% (19)*
Out of the top 20	–	64.3% (193)	77.0% (231)	88.0% (264)	91.0% (273)	93.7% (281)
Transition rate (remaining top 20)	–	35.7%	55.1%	47.8%	61.1%	66.7%

* Significant change compared to the previous category (*p* < 0.05)

Table 2 shows retrospective analysis of the top 20 senior athletes. From the total (64), most of them were already ranked top 20 at the U16 (59.4%, 38), U18 (62.5%, 40), U20 (70.3%, 45) and U23 (78.1%, 50) category. Nevertheless, only 34.4% (22) were top 20 when they were U14 athletes. The top 20 transition rate (Table 2) from U14 to U16 was 95.5% (21) of athletes ranked top 20 in U14. In addition, 17 athletes (40.5% of athletes out of top 20 at U14) entered top 20 at the U16 category for the first time, making the change between these categories statistically significant (*p* < 0.001). In the subsequent age bands, the change between categories was not significant (*p* > 0.05 in all cases), with very similar transition rates (around 87%). First entry in top 20 occurred mainly in U14 (34.4%, 22) and U16 (26.6%, 17) categories, while the rest achieved top 20 for the first time in subsequent categories. Around one out of four athletes (26.6%, 17) maintained top 20 status throughout their sporting careers from U14 to the senior category.

**Table 2:** Retrospective analysis of jumpers ranked top 20 in the senior category with data from previous categories.

	U14	U16	U18	U20	U23	Senior
Top20	34.4% (22)	59.4% (38)*	62.5%(40)	70.3%(45)	78.1% (50)	64
Out of the top 20	65.6% (42)	40.6% (26)	37.5% (24)	29.7% (19)	21.9% (14)	–
Transition rate (remaining top 20)	–	95.5% (21)	86.8% (33)	87.5% (35)	86.7% (39)	100% (50)
First entry in the top20	34.4% (22)	26.6% (17)	10.9% (7)	10.9% (7)	6.3% (4)	10.9% (7)

* Significant change compared to the previous category (*p* < 0.05).

## Discussion

The aim of this study was to analyze the sporting progression from U14 to senior categories of elite high and long jumpers. Data from 300 athletes with early success (ranked top 20 at the U14 category) were prospectively analyzed and data from 64 top 20 senior athletes were retrospectively examined to describe the maintenance of the top-level status alongside the successive age categories. The main results of this descriptive study reflect that a minority of athletes ranked top 20 at an early age maintained this status in subsequent categories, and only 6.3% reached the top 20 status as senior athletes (Table 1). The transition rate from U14 to U16 was the biggest obstacle to keep top 20 status (Table 1). On the other hand, most of the top 20 senior athletes were already ranked top 20 in the previous categories, except in U14, and showed higher transition rates between categories (Table 2). The first appearance in the top 20 positions for these successful senior athletes, occurred mainly at an early age (61% already in U16).

As it has been previously reported (Agudo-Ortega et al., 2023; Boccia et al., 2017, 2021b; Kearney and Hayes, 2018), most of the top 20 athletes at early ages did not maintain the same status later in their sporting career, thus being a successful U14 athlete did not guarantee to remain in that privileged position at senior age. Prospective analysis (Table 1) revealed that only 6.3% (19) of athletes who reached the U14 top 20 maintained this status at the senior level, and only 4.7% (14) remained in the top 20 throughout their sporting careers from U14 to senior, highlighting the difficulty of maintaining high performance. Our data are in accordance with previous research on world-class jumpers (Boccia et al., 2021b) where only 8% of male and 16% of female jumpers ranked top 50 at the age of 16 (U18) remained top 50 at the senior level. Presumably, this figure would be even lower if U14 data were available. In the same way, Kearney and Hayes (2018) found that the retention of top 20 high and long jumpers from UK (U13 to U20 analysis) was between 10.0% and 13.9%. Our outcomes showed the retention rate of 12.0% in U20 top 20, but this figure dropped to 9.0% and 6.3% at U23 and senior categories, respectively. Although variables affecting this phenomenon are out of the topic of our research, it is obvious that transition from youth to adult success is a complex process and is characterized by a remarkable amount of uncertainty, where the quality of training and competition, access to better coaches or facilities, family support, injuries, psychological factors, and/or socioeconomic background, could mediate sporting performance (Barth et al., 2022; Hollings et al., 2014; Issurin, 2017; Rees et al., 2016).

In addition, the transition rate from U14 to U16 (35.7%) was the most severe drop in subsequent categories. In the same way, in retrospective analysis, while most of senior athletes ranked top 20 were also top 20 from U16 to U23, only 34.4% were top 20 when they were U14 athletes. Clearly, this age band (from U14 to U16) represents a key point in the sporting career. This could be explained by differences in growth and maturation at these early ages. Around the growth spurt, differences in biological maturation could be considerable and significantly affect functional capacities (Charbonnet et al., 2022; Javet et al., 2022). Early maturation elicits physiological, physical, and functional advantages that transfer directly into performance environments (Sweeney et al., 2023). Thus, the inter-individual maturity differences, added to the relative age effect (born earlier in the selection year), could be responsible for some early successful athletes. At early ages, the early maturing jumpers are favoured when competing with their slower maturing peers, but this advantage could be eliminated after adolescence (Charbonnet et al., 2022; Rees et al., 2016; Sweeney et al., 2023).

On the other hand, retrospective analysis showed that most of the top 20 senior athletes were already ranked top 20 in the previous categories (Table 2), from 78.1% at the U23 to 59.4% at the U16 category. The early success could be mediated by individual talent (e.g., genetic factors) and this is sustained over time and thus, favouring maintaining top 20 positions. In addition, early success could contribute to the so-called “push” factors, such as greater motivation and sport adherence, resulting in the decision to specialise in that particular sports discipline (Huxley et al., 2017).

This study presents some limitations. First, the time span of data available did not allow to obtain a larger sample. To include data from U14 to the senior category, only jumpers born between 1993 and 1996 could be considered. This limited the size of the sample, but allowed us to analyse the whole sporting progression. Future research will allow us to increase the sample size and confirm or refuse these data. Second, athletes changing athletic discipline were considered as drop out, and it may be possible that some of them may have remained at the top level in their new discipline (e.g., sprint or hurdles events). Finally, the reasons for status change remain unexplored. Athletes could be slowed down or drop out due to injuries, lower deliberate practice or biological maturation, among other factors. Longitudinal analysis with other research designs should be performed to consider these key variables in sports performance progression.

## Conclusions

Although early success is not a good predictor of senior success, most successful senior athletes excelled early on and were able to remain in top rankings throughout their sporting careers as national elite jumpers.
